# Socioeconomic inequality in cognitive impairment among India’s older adults and its determinants: a decomposition analysis

**DOI:** 10.1186/s12877-022-03604-4

**Published:** 2023-01-05

**Authors:** Madhurima Sharma, Manas Ranjan Pradhan

**Affiliations:** 1 International Institute for Population Sciences, Govandi Station Road, Deonar Mumbai, India; 2 Department of Fertility and Social Demography, International Institute for Population Sciences, Govandi Station Road, Deonar, Mumbai, India

**Keywords:** Older adults, Cognitive impairment, Concentration index, Decomposition analysis, India

## Abstract

**Background:**

Cognitive impairment (CoI) is a significant risk factor for ill-health status among the older adults and a major burden on public health. This study unearths the degree of socioeconomic inequalities and assesses the determinants of CoI among the older adults in India.

**Methods:**

Data on cognitive impairment of older adults aged 60 + years (*n* = 31,646) gathered in a nationally representative Longitudinal Ageing Study in India (2017–18) was analyzed through STATA with a significance level of 5%. Binary logistic regression, the concentration index, concentration curve, and Shapley decomposition analysis were performed to assess the socioeconomic inequalities and the determinants of CoI.

**Results:**

Sixteen percent of the older adults had CoI. Females (OR = 1.88, 95% CI = 1.70–2.09), those aged 80 plus years (OR = 3.98, 95%CI = 3.56–4.44), from ST (OR = 2.65, 95%CI = 2.32–3.02), with perceived poor health (OR = 1.61,95%CI = 1.45–1.79), with depression (OR = 1.32, 95%CI = 1.21–1.43), with no schooling (OR = 16.46, 95%CI = 11.31–23.97) with 1 + ADL (OR = 1.43, 95%CI = 1.31–1.57) and 1 + IADL (OR = 1.30, 95%CI = 1.19–1.41) had higher odds of CoI than their respective counterparts. Older adults from urban areas (OR = 0.63, 95%CI = 0.57–0.70), higher income groups (OR = 0.61, 95%CI = 0.53- 0.70) and higher education level with sources of financial support (OR = 0.68, 95%CI = 0.61- 0.76) less likely to experience CoI. Economic inequalities exist in the distribution of CoI-the poorest being the most disadvantaged (concentration index value = -0.118).

**Conclusions:**

There are socioeconomic-related inequalities in CoI among the older adults. The socioeconomically vulnerable older adults, including those illiterates, with poor economic status, women, not-in-union, the older, and those without social support, are more likely to develop CoI. The results suggest awareness generation and more customized policies and programs to reduce the socioeconomic inequalities in CoI among the older adults in India. The improved mental health of the older adults will contribute to achieving Sustainable Development Goals, including Goal 3 on guaranteeing good health and well-being for all.

## Introduction

The size of the elderly population is on the rise worldwide, including in India [1]. Rapid demographic aging is expected to increase the prevalence of various non-communicable diseases and disabilities [2]. Cognitive impairment (CoI) is a significant risk factor for ill-health status among the older adults and a major burden on the public health of any nation [3]. CoI is when a person has trouble remembering, learning new things, concentrating, or making decisions that affect his/her everyday life [4]*.* Worldwide, around 55 million people have dementia- a severe decline in cognitive function, with over 60% living in low- and middle-income countries [5]. In India, 20 per 1000 elderly aged 75 years and above have dementia [6]. Healthier cognitive capacity is associated with better mental and physical health and lower mortality [7]. Males perform much better than females in cognitive performance, even after adjusting for diverse socioeconomic, demographic, and health-related characteristics [8]. Education was the most widely studied among socioeconomic variables, with protective effects on cognitive function reported in different cultural and geographic contexts [9]. However, comparatively few studies have examined the influence of other socioeconomic factors such as income, wealth, and occupation on the cognitive abilities of the older adults. Contradictory findings across studies further limit our understanding of their role and importance.

With the aging population and the expected rise in older adults with CoI, there has been an increasing interest in studying the association between socioeconomic status and cognitive performance [10]. To the best of our knowledge, there is no empirical study based on nationally representative data assessing the CoI among the older adults in India. The present study intends to identify the prevalence of CoI and risk factors. Additionally, this study unearths the degree of socioeconomic inequalities and assesses the determinants of CoI among older adults aged 60 years and above in the Indian context.

## Methods

### Study population

This study used the Longitudinal Ageing Study in India (LASI-Wave-I), a nationally representative survey of 72,250 older adults aged 45 and above conducted in 2017–18. The survey followed a multistage stratified area probability cluster sampling design to arrive at the eventual units of respondents. The survey in rural areas adopted a three-stage sampling design, and a four-stage sampling design in urban areas. The first stage involved selecting Primary Sampling Units (PSUs) in each state and union territories, that is, sub-districts (Tehsils/Taluks), and the second stage involved the selection of villages in rural areas and the selection of wards in urban areas in the selected PSUs. In the third stage, households were selected from selected villages in rural areas. However, sampling in urban areas involved an additional stage: one Census Enumeration Block (CEB) was randomly selected in each urban area. In the fourth stage, households were selected from these CEBs. Each consenting respondent in the sampled households was administered an individual survey schedule. The Indian Council of Medical Research (ICMR) extended the ethical approval for conducting the LASI. All methods were carried out in accordance with relevant guidelines and regulations. Informed consent was obtained from all subjects and/or their legal guardian(s). The detailed methodology, with complete information on the survey design and data collection, ethical considerations, and quality control measures, is available in the published survey report [11]. For this study, the sample of older adults aged 60 + years were considered (*n* = 31,646; men = 16,366 and women = 15,098).

### Outcome variable

The outcome variable for this study was ‘cognitive impairment’, which was assessed using the composite cognition score based on five cognitive domains named memory, orientation, arithmetic, executive functioning skills, and object naming. The composite cognitive score ranges from 0 to 43; the higher the value of the score indicates higher cognitive ability. The lowest 10^th^ percentile measured poor cognitive functioning [12]*.*

### Predictor variables

*Demographic factors* included were gender (male, female), age (60–69, 70–79, 80 + years), marital status (currently-in-union, not-in-union), caste (scheduled caste-SC, scheduled tribe-ST, other backward classes-OBC, and none of them), religion (Hindu, others), place of residence (rural, urban), and region (north, west, south, east, and north-east). The s*ocioeconomic factors* considered were years of schooling (no schooling, up to 9 years, and 10 and more years), working status (currently working and currently not working), and monthly per capita consumption expenditure (MPCE quintile). T*wo element*s assessed the social support: financial support (no, yes) and living arrangements (living alone, living with spouse and/or others, living with spouse and children, living with children and others, and living with others only). *Health aspects* included as predictors were body mass index (BMI- underweight, normal, obese), self-rated health (good, moderate, poor), depression (no, yes), difficulty in activities of daily living (ADL- no, yes), and instrumental activities of daily living (IADL- no, yes). The CES-D scale [13] was used to estimate the presence of depressive symptoms. Additionally, alcohol consumption (yes, no) and smoking (yes, no) were considered under the health aspects.

### Statistical analysis

The Stata 16.1 software was used for the data analysis. An analysis of the differences was conducted using the *Chi-square test*. The sample population was classified into two groups according to their level of cognitive function as per composite cognitive score: (i) 0 represents ‘do not have cognitive impairment’ and (ii) 1 represents ‘Have cognitive impairment’. In first stage, Multiple binary logistic regression analysis was used to estimate the effects of the demographic, socioeconomic, social support, and health factors on CoI.

The equation for logistic distribution is:

$$l_n\frac{\mathrm\pi}{\left(1-\mathrm\pi\right)}\;=\;a+\beta_1X_1+\beta_2X_2+\beta_3X_3+\dots+\beta_nX_n$$

where, *X*_*1*_*, X*_*2,*_* X*_*3*_*,…X*_*n*_ are explanatory variables and *β*_*1*_*, β*_*2*_*, β*_*3*_*, … β*_*n*_ are regression coefficients.

In second stage, *the concentration index (C)* and *concentration curve* (CC) were prepared to reflect the expense-related economic inequality in CoI. The present study examined CoI among the older adults by economic status quintiles. The C was defined as twice the area between the line of equality and CC. The CC was plotted based on the cumulative percentage of CoI on the Y-axis against the cumulative percentage of the population ordered by economic status on the graph's X-axis. The C can be calculated using the following formula:

$$\text{Concentration index}\left(\text C\right)=\frac2{\mathrm\mu\;\boldsymbol\ast\boldsymbol\;cov(h,r)}$$

where, h = the health outcome (CoI among older adults in the study) $\upmu$ = the mean of h, r = the fractional rank of individuals in the distribution used (economic status quintiles). The value of the C ranges between -1 to + 1, a value of '0' represents absolute equality or fairness, and there is no income-related inequality in terms of CoI. A positive C value indicates that CoI is more concentrated among richer people (pro-poor), while a negative value suggests more concentration among poor people (pro-rich).

To determine the impact of the different categories of explanatory variables, we used the concept of Shapley decomposition [14–16] which is quite well known. We applied the simplest type of Shapley decomposition to determine the impact of demographic, socioeconomic, social support, and health-related variables on the inequality of the CoI among the older adults. The Shapley value decomposition is useful in regression-based methods as it does not require the regression model to be linear. The Shapley value decomposition method relies on iteratively removing explanatory variables to determine how much each contributed to overall inequality. It should be emphasized that we describe the process using the zero Shapley decomposition technique. To perform the Shapley value decomposition analysis, we have included four categories of variables reflecting demographic, socioeconomic, social support, and health factors. Demographic variables include sex, age, marital status, religion, and place of residence of the respondents; socioeconomic variables consist of education level and wealth status (MPCE quintile) of the respondents; social support variables include financial support and living arrangements; and lastly, health-related variables include self-rated health, alcohol consumption status, ever smoked status, difficulties in ADL and IADL and depression. A grouped Shapley decomposition has been performed to reflect the impact of variables on the inequality of CoI. we take into account all possible combinations of demographic, socio-economic, social support and health factors via the so-called Shapley decomposition procedure.

*d*_*ki*_ denotes the level of the circumstance variable k (k = 1,…, K) for individual i, *e*_*hi*_ (h = 1,…, H) the socio-economic variable of individual i and *s*_*li*_ denotes the social support factors l (i = 1,…,L) for individual i. Finally, let us call *h*_*m*i_ the value of the health variable m (m = 1,…,M) for individual i.

The actual likelihood ratio can be written as,

$$LRI_1=LRI\left(dki\neq0;ehi\neq0;sli\neq0;hmi\neq0\right).$$

Assume for example that we do not include the demographic variables, d_ki_, of the different individuals in the regression in such a case the likelihood ratio will be expressed as,

$${\mathrm{LRI}}_2=LRI\left(dki=0;ehi\neq0;sli\neq0;hmi\neq0\right).$$

Similarly, assume that we do not include in the regression the socio-economic variables, *e*_*hi*_*.* In such a case we will define the likelihood ratio as, *LRI*_*3*_ = *LRI (d*_*ki*_ ≠ *0; e*_*hi*_ = *0; s*_*li*_ ≠ *0; h*_*mi*_ ≠ *0)*. We can also assume that we do not introduce in the regression the social support variables, *s*_*li*_*,* in which case the likelihood ratio will be *LRI*_*4*_ = *LRI (d*_*ki*_ ≠* 0; e*_*hi*_ ≠ *0; s*_*li*_ = *0; h*_*mi*_ ≠ *0).* Lastly, we assume that we do not include the heath factors in the regression then the likelihood ratio will be *LRI*_*5*_ = *LRI (d*_*ki*_ ≠ *0; e*_*hi*_ ≠ *0; s*_*li*_ ≠ *0; h*_*mi*_ = *0).*

Naturally, we could also decide not to include two sets of explanatory variables (e.g., the demographic and the socio-economic variables, the demographic and social support variables, the demographic and health factors, the socio-economic and social support variables, the socio-economic and health factors, the social support and health variables, named respectively, *LRI*_*6*_*, LRI*_*7*_*, LRI*_*8*_*, LRI*_*9*_*, LRI*_*10*_*, LRI*_*11*_*.*

Using the by now well-known Shapley procedure we derive that the contribution of demographic variables, **C**_**d**_ to the overall actual likelihood ratio, *LRI*_*1*_, may be expressed as,

${\mathbf C}_{\mathbf d}=\left(\frac28\right)\left(LRI_1-LRI_2\right)+\left(\frac18\right)\left(LRI_4-LRI_6\right)+\left(\frac18\right)\left(LRI_3-LRI_5\right)+\left(\frac18\right)\left(LRI_8-LRI_{10}\right)+\left(\frac18\right)\left(LRI_7-LRI_9\right)+\left(\frac28\right)\left(LRI_{11}\right)$, since by definition, LRI (*d*_*ki*_ = 0; *e*_*hi*_ = 0; *s*_*li*_ = 0; *h*_*mi*_ = 0) = 0.

Similarly, the contribution of socio-economic factors, **C**_**e**_, to the actual likelihood ratio, LRI_1_, may be expressed as,

$${\mathbf{C}}_{\mathbf{e}} =\left(\frac{2}{8}\right) \left(LR{I}_{1}-LR{I}_{3}\right) + \left(\frac{1}{8}\right) \left(LR{I}_{4}-LR{I}_{7}\right)+ \left(\frac{1}{8}\right) \left(LR{I}_{2}-LR{I}_{5}\right)+ \left(\frac{1}{8}\right) \left(LR{I}_{6}-LR{I}_{9}\right)+ \left(\frac{1}{8}\right) \left(LR{I}_{7}-LR{I}_{10}\right)+ \left(\frac{2}{8}\right) \left(LR{I}_{8}\right)$$

Likewise, the contribution of social support, **C**_**s**_, to the actual likelihood ratio, LRI_1_, may be expressed as,

$$\mathbf{C}_\mathbf{s}=\left(\frac28\right)\left(LRI_1-LRI_4\right)+\left(\frac18\right)\left(LRI_3-LRI_7\right)+\left(\frac18\right)\left(LRI_2-LRI_6\right)+\left(\frac18\right)\left(LRI_5-LRI_9\right)+\left(\frac18\right)\left(LRI_8-LRI_{11}\right)+\left(\frac28\right)\left(LRI_{10}\right).$$

Finally, the contribution of health-related variables, **C**_**h**_, to the actual likelihood ratio, LRI_1_, may be expressed as,

$${\mathbf C}_{\mathbf h}=\left(\frac28\right)\left(LRI_1-LRI_5\right)+\left(\frac18\right)\left(LRI_2-LRI_8\right)+\left(\frac18\right)\left(LRI_3-LRI_{10}\right)+\left(\frac18\right)\left(LRI_4-LRI_7\right)+\left(\frac18\right)\left(LRI_6-LRI_7\right)+\left(\frac28\right)\left(LRI_9\right)$$

It is then easy to verify that,

$$C_d+C_e+C_s+C_h=LRI\left(dki\neq0;ehi\neq0;sli\neq0;hmi\neq0\right)$$

In other terms, by taking into account all possible combinations of the sets of explanatory variables we can then easily derive the respective contributions of demographic variables, socio-economic, social support and health related variables to the actual likelihood ratio.

## Results

### Socioeconomic and demographic differentials in CoI

Sixteen percent of the older adults had CoI (Table 1). Among the males, 10%, and among females, 22% had CoI. The prevalence of CoI was higher among older adults individuals aged 80 + years (36%) than those aged 60–69 years (11%). A higher percentage (24%) of the older adults not-in-union had CoI than those in the union (11%). Thirty-one percent of the STs had CoI. The corresponding figure was 19% among the SCs, 15% among the OBC, and 12% among the general castes. The prevalence of CoI was higher among rural residents (18%) than their urban counterparts (11%). One-fifth of the older adults from the north-eastern region had CoI. Of those underweight, 12% had CoI, while 3% of those overweight/obese older adults had CoI. Twenty-one percent of those who perceived their health status as poor had CoI. A higher percentage of the older adults with 1 + ADL (28%) and 1 + IADL (23%) had CoI than their counterparts. The older adults with 10 or more years of schooling had a lower prevalence of CoI (5%) than those without schooling (24%). Elderlies who were not currently working had a higher prevalence of CoI (19%) than those currently working (9%). Twenty-one percent of the older adults from the poorest MPCE quintile against 12% from the richest quintile had CoI. Only 9% of the older adults living with spouses and children had CoI. The corresponding figure was 29% among those living with others only.

**Table 1:** Percentage of older adults (60 and above years) with CoI by background characteristics, India, 2017–18

Variables	Prevalence of CoI	n	chi square ( χ2)
	**%**		
**Demographic Factors**			
**Gender**			
Male	10.02	14,931	< 0.001
Female	21.76	16,533	
**Age (in years)**			
60–69	10.66	18,410	< 0.001
70–79	19.42	9501	
80 + years	36.22	3553	
**Marital Status**			
Currently-in-union	11.23	19,391	< 0.001
Currently-not-in-union	24.16	12,073	
**Social groups**			
SC	18.89	5906	< 0.001
ST	30.51	2537	
OBC	14.53	14,129	
None of Them	12.29	7844	
**Religion**			
Hindu	15.34	25,870	< 0.001
Others	20.1	5593	
**Place of Residence**			
Rural	18.2	22,196	< 0.001
Urban	11.38	9268	
**Region**			
North	14.79	3960	< 0.001
West	17.58	5401	
East	17.31	7439	
South	15.47	7136	
Central	14.92	6593	
North East	19.58	935	
**Health Factors**			
**BMI**			
Underweight	11.73	11,222	< 0.001
Normal Weight	5.73	10,616	
Overweight or Obese	3.24	6212	
**Self-rated health**			
Good	9.47	9449	< 0.001
Moderate	13.12	13,892	
Poor	21.13	7457	
**Depression**			
No	11.44	21,211	< 0.001
Yes	18.52	9178	
**Difficulty in ADL**			
No	12.51	24,042	< 0.001
1 + ADL difficulties	28.11	7422	
**Difficulty in IADL**			
No	10.13	16,370	< 0.001
1 + IADL difficulties	22.76	15,094	
**Alcohol Consumption**			
Yes	12.41	4555	< 0.001
No	15.85	26,655	
**Smoking**			
Yes	14.53	12,539	*P* = 0.037
No	15.89	18,665	
**Socio-Economic Status**			
**Years of schooling**			
No schooling	24.35	17,783	< 0.001
Up to 9 years	5.89	9209	
10 and more years	4.96	4472	
**Working status**			
Currently working	9.34	9483	< 0.001
Currently Not working	19.02	13,197	
**MPCE quintile**			
Poorest	20.93	6829	< 0.001
Poorer	18.27	6831	
Middle	15.18	6590	
Richer	13.19	6038	
Richest	11.97	5175	
**Social Support**			
**Financial Support**			
Yes	15.29	4713	*P* = 0.075
No	15.19	26,266	
**Living arrangements**			
Living alone	22.15	1787	< 0.001
Living with spouse and/or others	15.07	6397	
Living with spouse and children	9.07	12,779	
Living with children and others	23.55	8696	
Living with others only	29.2	1805	
**Total**	**15.61**	**31,464**	

### Determinants of CoI

Except for marital status, religion, alcohol consumption, smoking status, and living arrangements, all other factors considered in the regression model were predicted to influence CoI significantly (Table 2). Females had higher odds of CoI (OR = 1.88, 95% CI = 1.70–2.08) compared to males. Compared to the older adults aged 60–69 years, those aged 80 years or above were 3.9 times more likely to have CoI (OR = 3.98, 95%CI = 3.56–4.44). The chances of CoI were higher for the older adults from OBC (OR = 1.06, 95% CI = 0.94–1.18) and ST (OR = 2.65, 95CI% = 2.32–3.02) categories than those from SCs. The older adults from the urban areas were less likely to suffer from CoI than their rural counterparts (OR = 0.63, 95%CI = 0.57–0.70). The older adults perceived to have poor health status had higher odds of CoI than those with perceived good health (OR = 1.61,95%CI = 1.45–1.79). Depressed elderlies had a higher likelihood of CoI than the non-depressive group (OR = 1.32, 95%CI = 1.21–1.43). The older adults with 1 + ADL (OR = 1.43, 95%CI = 1.31–1.57) and 1 + IADL (OR = 1.30, 95%CI = 1.19–1.41) had higher chances of CoI than their respective counterparts. As compared to the respondents who had attended 10 years of schooling and more, those who had not attended school ever had higher odds of CoI (OR = 16.46, 95%CI = 11.31—23.97), and those who attended up to 9 years of schooling had around 3% higher odds of CoI (OR = 3.94, 95%CI = 2.68- 5.78) which is comparatively lower than uneducated elderlies. Higher income was linked to a lower likelihood of CoI. For example, compared with the poorest, the poorer, middle, richer and richest (OR = 0.61, 95%CI = 0.53- 0.70) groups had a lower probability of CoI. Older adults having sources of financial support from family or friends were at lower risk (OR = 0.68, 95%CI = 0.61- 0.76) of CoI.

**Table 2:** Binary logistic regression of determinants of CoI among Older adults (60 and above) India, 2017–18

Variables	OR	CI (95%)	
		**lower**	**Upper**
**Demographic Factors**			
**Gender**			
Male (ref)			
Female	1.88***	1.70	2.09
**Age (in years)**			
60–69 (ref)			
70–79	1.75***	1.60	1.91
80 + years	3.98***	3.57	4.45
**Marital Status**			
Currently-in-union (ref)			
Currently-not-in-union	1.09	0.71	1.69
**Social groups**			
SC	1.21**	1.06	1.37
ST	2.65***	2.32	3.03
OBC	1.06	0.95	1.19
None of them (ref)			
**Religion**			
Hindu (ref)			
Others	1.06	0.96	1.16
**Place of Residence**			
Rural (ref)			
Urban	0.64***	0.58	0.70
**Region**			
North (ref)			
West	1.54***	1.34	1.77
East	0.99	0.87	1.13
South	0.91	0.80	1.03
Central	0.74***	0.65	0.86
North East	0.96	0.82	1.13
**Health Factors**			
**Self-rated health**			
Good (ref)			
Moderate	1.13*	1.02	1.24
Poor	1.62***	1.45	1.80
**Depression**			
No (ref)			
Yes	1.32***	1.2	1.43
**Difficulty in ADL**			
No (ref)			
1 + ADL difficulties	1.44***	1.31	1.58
**Difficulty in IADL**			
No (ref)			
1 + IADL difficulties	1.30***	1.19	1.42
**Alcohol Consumption**			
Yes (ref)			
No	0.90	0.80	1.02
**Smoking**			
Yes (ref)			
No	0.98	0.90	1.07
**Socio-Economic Status**			
**Years of schooling**			
No schooling (ref)	16.47***	11.31	23.97
Up to 9 years	3.94***	2.69	5.78
10 and more years			
**MPCE Quintile**			
Poorest (ref)			
Poorer	0.88*	0.79	0.98
Middle	0.76***	0.67	0.85
Richer	0.64***	0.56	0.72
Richest	0.61***	0.54	0.70
**Social Support**			
**Financial Support**			
Yes	0.69***	0.62	0.77
No (ref)			
**Living arrangements**			
Living alone (ref)			
Living with spouse and/or others	0.84	0.53	1.33
Living with spouse and children	0.77	0.49	1.22
Living with children and others	1.04	0.89	1.21
Living with others only	1.14	0.94	1.39
**Constant**	0.09***	0.06	0.15
**Log-likelihood**	-8937.93		
**Prob > chi2**	0.000		

### Measurement of Socioeconomic inequalities in CoI

**Fig. 1:**
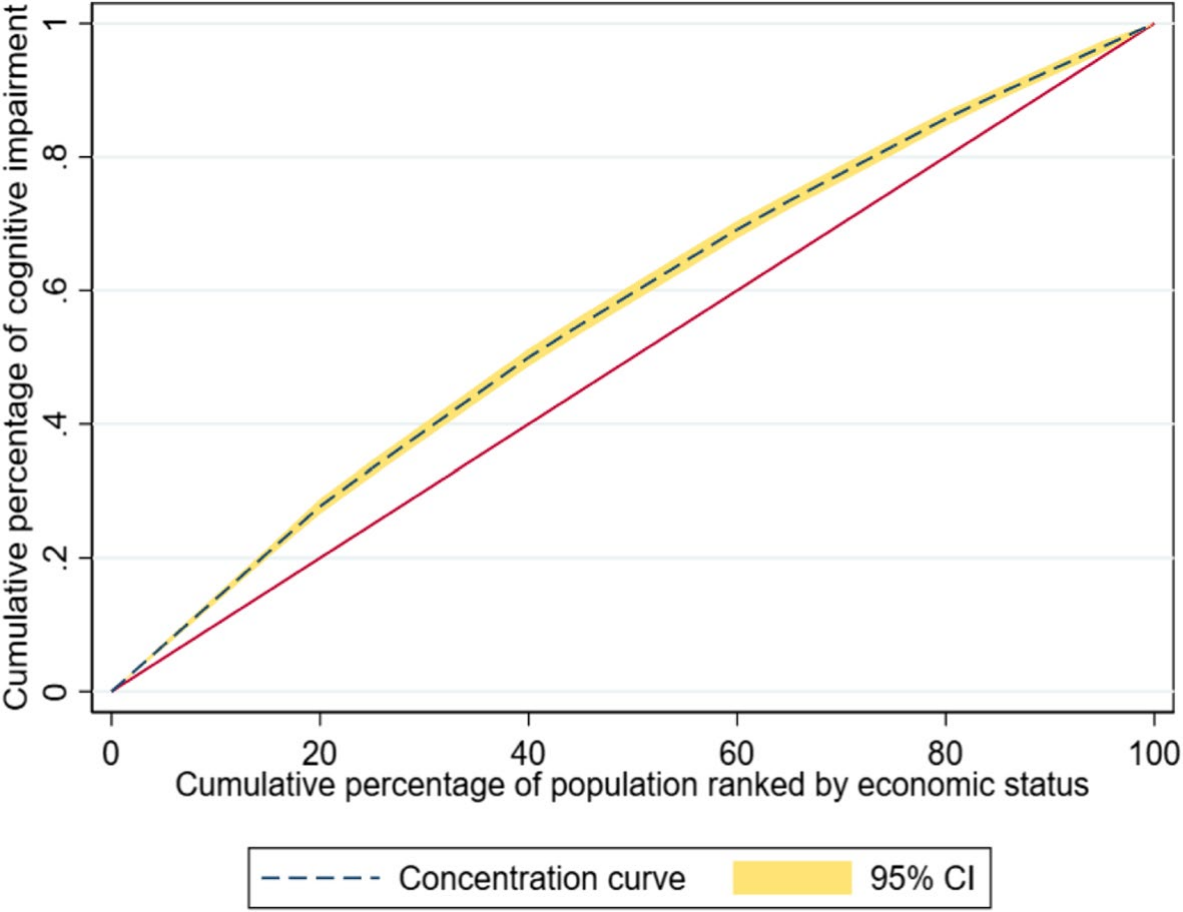
Concentration curve of cognitive impairment

The CC of CoI lies above the line of equality (Fig. 1), with a negative value of the C of -0.118 (Table 3). This indicates that economic inequalities exist in the distribution of CoI. Moreover, the inequalities are disadvantageous to the poor.

**Table 3:** Tabulation of Concentration Index of Cognitive Impairment based on socio-economic status among the older adults in India 2017–18

Index	No. of obs	Index value	Std. error	*p*-value
CI	31,464	-0.11841602	0.00722334	0

### Results of the Shapley decomposition analysis

Tables 4 and 5 present the results of the Shapley decomposition. The models explain the socioeconomic inequalities in CoI among the older adults in India (Table 4). The results revealed that the demand-side factors include wealth index for households, education of the older adults, age and sex of the respondent, and other demographic background contribute the largest portion to income-related inequality in CoI among elderlies. On the other hand, the supply-side factors such as financial support, living arrangement, and other social support factors contribute least to the total inequality. Next, we estimated the contributions of various factor groups to the inequality using the Shapley decomposition given in the equation. Results (Table 5) showed that demographic and socioeconomic factors contribute the largest portion to socioeconomic inequality of CoI. In particular, in the case of a binary CoI outcome, demographic variables explain 41%, socioeconomic variables 45%, social support factors 3%, and health-related variables 14% of the likelihood ratio. In other words, the results depict the largest contribution of the socioeconomic variables to the income-related inequalities of CoI among the older adults, followed by the demographic variables, which also significantly impact the inequality measurement of CoI.

**Table 4:** Contributions of the predictor variables to the Pseudo R-square of the cognitive impairment among older adult population logit regression

Contributors	Marginal effect (dy /dx)	*P* value	Absolute Contribution	Relative Contribution (in %)
Gender	0.044	0.000	0.022	11.1
Age (in years)	0.037	0.000	0.037	18.78
Marital Status	0.008	0.000	0.017	8.8
Place of Residence	-0.032	0.000	0.012	6.2
Depression	0.018	0.000	0.005	2.37
Difficulty in ADL	0.047	0.000	0.011	5.81
smoke ever	-0.007	0.000	0.001	0.31
Years of Schooling	-0.102	0.000	0.082	41.97
MPCE Quintile	-0.007	0.000	0.008	4.08
Financial support	-0.009	0.000	0.001	0.43
Living arrangements	0.001	0.000	0.004	2.07
**Pseudo R-square**	**0.195**			**100**

**Table 5:** Contributions of the demographic, socio-economic, social support, and the health related variables to the Pseudo R-square of the cognitive impairment logit regression

Contributors	Demographic variables	Socio-economic variables	Social support variables	Health-related variables	Total
Absolute Contribution	0.082	0.089	0.006	0.028	0.203
Relative Contribution (in %)	41.22	44.59	3.15	14.13	100

## Discussion

A sizable number of older adult people suffer from CoI, and the prevalence varies considerably by socioeconomic and demographic characteristics. The CoI is mainly concentrated among the older adults with lower socioeconomic status- uneducated and economically disadvantaged. The study found females at a higher risk of CoI, which is in line with previous studies conducted in India [17]. The possible reason may be gender discrimination, limiting women's access to education and financial resources [18]*.* We found increasing age as a significant risk factor for CoI, which agrees with a past study[19]*.* We found a lower prevalence of CoI in urban areas, possibly due to socioeconomic factors playing an essential role in cognitive function in urban areas [20]. An earlier Indian study also reported rural–urban differences in cognitive function among older adults in India [21]*.* Depression was found to be associated with CoI. This result conforms with a recent study [22]*.* Our results suggest that the older adults with no ADL difficulty have higher cognitive scores. The finding is consistent with previous studies that concluded that interventions that promote higher levels of physical activity in old age are associated with a slower rate of cognitive decline among the older adults [19, 23]. This study's findings are also consistent with earlier research that supports the links between personality, self-rated health, and cognitive performance in older adults [24, 25]. We found financial support as a protective factor against CoI*. *Evidence suggests that access to financial support can help them prevent low cognitive conditions, mainly after retirement [26].

The decomposition analyses suggest that socioeconomic variables (wealth status of household, education level of respondents) contribute the largest to the total inequality in CoI among the older adults. Demographic variables also play a significant role in explaining inequality. Studies in developing and developed countries [27, 28] have implied how the socioeconomic status of individuals affects their cognition by the exposure to the resources available and the environment. Moreover, the study found higher concentration of CoI among the poor, which conforms to previous studies in China and India [3, 29].

There are several strengths of this study. It is the first study to analyse the socioeconomic inequality in CoI besides the determinants of CoI among the older adults. The study uses the recent large-scale nationally representative LASI data with a robust sampling design; thus, the results are contemporary and relevant. Nevertheless, the cross-sectional data used for analysis limits the inferences drawn on the causal association between the predictors and outcome variables.

## Conclusion

There are socioeconomic-related inequalities in CoI among the older adults in India. The socioeconomically vulnerable older adults, including those illiterates, with poor economic status, women, non-in-union, the older, and those without social support are more likely to suffer from CoI. The results suggest awareness generation and more customized policies/programs to reduce the socioeconomic inequalities in CoI. The proportion of older adults in the Indian population is increasing, and the size of the older adults with CoI is also expected to increase. Hence, efforts to acquaint the disadvantaged older adults with the role of modifiable lifestyle factors in CoI seem pertinent. Moreover, preventive interventions such as early identification and appropriate treatments would improve cognitive performance and prevent progressive impairment. The improved mental health of the older adults will also contribute to achieving Sustainable Development Goals (SDGs), including Goal 3, guaranteeing good health and well-being for all.

## Data Availability

The datasets generated and/or analysed during the current study are available in the [IIPS] repository, [https://iipsindia.ac.in/content/data-request].
